# Population level mental distress in rural Ethiopia

**DOI:** 10.1186/1471-244X-14-194

**Published:** 2014-07-07

**Authors:** Abebaw Fekadu, Girmay Medhin, Medhin Selamu, Maji Hailemariam, Atalay Alem, Tedla W Giorgis, Erica Breuer, Crick Lund, Martin Prince, Charlotte Hanlon

**Affiliations:** 1 Department of Psychiatry, School of Medicine, College of Health Sciences, Addis Ababa University, PO Box 9086, Addis Ababa, Ethiopia; 2 King’s College London, Institute of Psychiatry, Department of Psychological Medicine, Centre for Affective Disorders, London, UK; 3 Addis Ababa University, Aklilu Lemma Institute of Pathobiology, Addis Ababa, Ethiopia; 4 Federal Ministry of Health, Addis Ababa, Ethiopia; 5 Alan J Flisher Centre for Public Mental Health, Department of Psychiatry and Mental Health, University of Cape Town, Cape Town, South Africa; 6 King’s College London, Institute of Psychiatry, Health Services and Population Research Department, London, UK

**Keywords:** Common mental disorders, Psychosocial distress, Mental distress, Suicidality, Hazardous alcohol use, Wellbeing, Developing country, Africa South of the Sahara, Sub-Saharan Africa, Ethiopia

## Abstract

**Background:**

As part of a situational analysis for a research programme on the integration of mental health care into primary care (Programme for Improving Mental Health Care-PRIME), we conducted a baseline study aimed at determining the broad indicators of the population level of psychosocial distress in a predominantly rural community in Ethiopia.

**Methods:**

The study was a population-based cross-sectional survey of 1497 adults selected through a multi-stage random sampling process. Population level psychosocial distress was evaluated by estimating the magnitude of common mental disorder symptoms (CMD; depressive, anxiety and somatic symptoms reaching the level of probable clinical significance), harmful use of alcohol, suicidality and psychosocial stressors experienced by the population.

**Results:**

The one-month prevalence of CMD at the mild, moderate and severe threshold levels was 13.8%, 9.0% and 5.1% respectively. The respective one-month prevalence of any suicidal ideation, persistent suicidal ideation and suicide attempt was 13.5%, 3.8% and 1.8%. Hazardous use of alcohol was identified in 22.4%, significantly higher among men (33.4%) compared to women (11.3%). Stressful life events were widespread, with 41.4% reporting at least one threatening life event in the preceding six months. A similar proportion reported poor social support (40.8%). Stressful life events, increasing age, marital loss and hazardous use of alcohol were associated with CMD while stressful life events, marital loss and lower educational status, and CMD were associated with suicidality. CMD was the strongest factor associated with suicidality [e.g., OR (95% CI) for severe CMD = 60.91 (28.01, 132.48)] and the strength of association increased with increase in the severity of the CMD.

**Conclusion:**

Indicators of psychosocial distress are prevalent in this rural community. Contrary to former assumptions in the literature, social support systems seem relatively weak and stressful life events common. Interventions geared towards modifying general risk factors and broader strategies to promote mental wellbeing are required.

## Background

Common mental disorders (CMD) refer to either the occurrence of a combination of non-specific anxiety, depressive and somatic symptoms [1] or anxiety, depressive and somatoform disorders “usually measured” with screening tools [2]. The exact genesis of the term is not clear but appears to have come into use with the decline in the use of the term “neurosis”. The leaders in the study of CMD have understood the shortcomings of the current international classification systems [3]. They argue that most non-psychotic disorders have poorly defined boundaries and most individuals presenting to a primary care are likely to have a combination of anxiety, cognitive, depressive, somatic and vegetative complaints. Therefore, the term CMD has relevant heuristic value. But by using the term “common” to signify the common-ness of the CMDs, CMDs are often viewed as trivial and transient. Most descriptions of CMD are limited to the description of the mixed phenomenology and risk factors. Their treatment and course are not fully explored because the concept does not inform treatment to the satisfaction of clinicians. CMDs also do not fully match to the current international nosological systems of the International Classification of Diseases (ICD-10) [4] or the Diagnostic and Statistical Manual of Mental Disorders [5].

However, the CMD construct may be a very useful measure of the mental wellbeing of a population or a community, particularly if the assessment explores additional psychosocial risk factors that may indicate population level wellbeing and risk. CMDs can be measured easily and with minimal cost using a broad range of brief instruments that can be administered by lay interviewers with limited training. Because of the nature of categorical disorders defined by DSM and ICD systems, the level of mental disorder in a population is likely to be underestimated and, by measuring CMDs, the overall level of the psychosocial distress of a population could be evaluated more accurately resulting in an estimation closer to the actual population level morbidity. Additionally co-morbidity is less of an issue although substance related conditions are more discrete and need a separate measurement. Finally CMDs may predispose to more serious disorders although providing services may be more challenging. Therefore CMDs are likely to account for the majority of the burden of mental disorder in a population. This makes CMD of public health relevance and may be meaningful constructs to public health specialists and policy makers. CMDs should also inform treatment and attract service structuring and investment [3].

Additional population level indicators of mental distress and wellbeing may be suicidal behaviour and violence, and substance abuse. Suicidality is an important dimension of mental distress, which may be taken as an indication of the severity of a mental disorder. Suicidality may also be closely linked with impulse control, but probably mediated through various stressors and life events. Moreover, information on suicidal behaviour may not be volunteered by patients or families in low income countries because of the strong negative attitudes and shame associated with the behaviour in these settings [6-8]. Related to this negative attitude, service utilisation may be compromised. Thus, suicidal behaviour, CMD and other related psychosocial problems are important public health concerns that require further exploration. The main objective of this study was to explore the population level of burden of psychosocial problems in a rural community in Ethiopia. Specifically we aimed to determine the burden of CMD and suicidal behaviour and to assess for potential psychosocial and demographic factors associated with these outcomes.

The study was conducted as part of a situational appraisal for the Programme for Improving Mental Healthcare (PRIME) [9]. PRIME is a cross-country research consortium involving five low and middle income countries (Ethiopia, India, Nepal, South Africa and Uganda). The primary aim of PRIME is to develop evidence on the best methods of integrating mental health care into primary care. PRIME in Ethiopia will introduce broad community based interventions and facility based interventions to support integration. The current baseline survey is an important first step to understand the baseline mental health context of the community in the study setting.

## Methods

The study was a cross-sectional survey of adults aged 18 years and above. The study participants were selected randomly from all sub-districts of the Sodo district proportional to the size of the population of each sub-district.

### Setting

The study was conducted in the Sodo district, Gurage Zone, Southern Nations, Nationalities and Peoples Region (SNNPR), a predominantly rural district located about 100 km south of the capital city, Addis Ababa. The population of the district is 161,952 persons (79,356 men; 82,596 women) living in 58 subdistricts (*kebeles)*[10]. The largest ethnic group in the district is Sodo Gurage (85.3%) followed by Oromo (11.6%) and Amhara (1.5%) and Amharic is the official language. The majority of the population are Orthodox Christian (97%) with Muslims making up 2.3%. Within Sodo district there are seven public health centres and one health centre run in a public-private partnership. There are 54 health posts (community based facilities), with another two under construction. The nearest hospital is located in Butajira town, 30 km South of Bui town, the capital of the district. At present there is no formal mental health care provided within the district. The nearest service is the nurse-led psychiatric unit in Butajira hospital. Sodo district was selected for this project because it is a relatively typical rural district for Ethiopia, and is located close to the research infra-structure of the Butajira research project on severe mental disorders and the Butajira Demographic Surveillance Site [11,12]. The site is also within reasonable travel distance of specialist mental health services.

### Participants

Participants were consenting adults, aged 18 years and above, who had been residing in the district for at least six months. Participants were selected through systematic random sampling of households within each sub-district and by random sampling of one adult from each selected household. The number of participants selected from each sub-district was allocated proportionate to the number of households within each sub-district. A total of 1497 participants were included in the study. This sample size was based on the assumption that the prevalence of CMD would be about 10%, with a design effect of 1.5 (due to the multistage sampling of study participants), a precision of 0.02 and a 15% non-response. The prevalence of 10% for calculating the sample size is based on a conservative approximation of the prevalence of CMD, which ranges from about 6% to about 40% (Table 1).

**Table 1:** Studies of CMD in Ethiopia over the past 40 years

**Reference**	**Location**	**Setting**	**SS**	**CMD measure**	**CMD definition**	**Prevalence**
**First Generation studies**						
[76]	Urban	Urban health centre	500	Psychiatrist	CPM	19.0%
	Urban	Community	100	Psychiatrist	CPM	8.6%
[77]	Rural	Community	100	Psychiatrist	CPM	9.0%
[78]	Urban	General hospital clinic	795	Psychiatrist	CPM	6.8%
	Urban	Police Hospital clinic	486	Psychiatrist	CPM	16.2%
[79]	Urban	District hospital clinic	465	Psychiatrist	CPM	18.3%
**Second Generation Studies**						
[80]	Urban	Community	40	SRQ-20	Cut-off ≥5	12.0%
[38]	Urban	Community (Mothers only)	611	SRQ-20	Cut-off ≥11	9.8%
[39]	Rural	Community	2000	SRQ-20	Cut-off ≥11	11.2%
[33]	Rural	Community	10468	SRQ-20	Cut-off ≥11	17.4%
[37]	Urban	Community	10203	SRQ-20	Cut-off ≥6	11.7%
[81]	Mixed	Community (Mothers only)	1400	SRQ-20	Cut-off ≥8	22.0%
[34,36]	Mixed	Community (Mothers only)	1652	SRQ-20	Cut-off ≥8	32.0%
[35]*	Rural	Community	902	HSCL		42.0% in women, 37.0% in men
[82]	Rural	Community (antenatal)	1065	SRQ-20	Cut-off ≥5	12.0%
[1]	Rural	Community (postnatal)	954	SRQ-20	Cut-off ≥5	4.6%
**Third Generation Studies**						
[41]	Rural	Community	501	CIDI 1-month	Dissociative disorders	4.5%
					Somatoform disorders	4.8%
					Anxiety disorders	2.9%
					Depressive disorder/dysthymia	4.9%
[46,47]	Urban	Community	1420	CIDI 1-month	Dissociative, somatoform or anxiety disorder	8.1%
			1420	CIDI 1-month	Depressive disorder/dysthymia	3.6%
[42,43]	Rural	Community (all women)	3016	CIDI 12-month	Depressive disorder	4.4%
					Anxiety disorder	5.7%
					Stress-related/somatoform	5.7%
		Community (married women)	1994	CIDI (12-month)	Depressive disorder	4.8%
[44]	Rural	Community	68378	CIDI (lifetime)	Minor depressive disorder	2.2%
[45]	Rural Island	Community	1714	CIDI (lifetime)	Minor depressive disorder	20.5

*Abbreviations:CIDI* Composite International Diagnostic Interview, *CMD* Common Mental Disorder, *CPM* Conspicuous Psychiatric Morbidity, *HSCL* Hopkins Symptom Checklist, *SRQ* Self Reporting Questionnaire.

### Assessment of CMD and psychosocial factors

The main outcomes of interest were CMD and suicidality. Suicidality was defined as a composite of persistent death wish, suicidal ideation and suicide attempt. Assessment instruments were administered by trained community health workers with a focus on evaluating demographic status, CMD, suicidality, alcohol use disorder and psychosocial stressors. Socio-demographic assessment established basic demographic characteristics (age, sex, marital status, religion, ethnicity) and socio-economic status (education, relative wealth and occupation). Relative wealth was assessed by simply asking the respondent what he/she perceived their wealth to be in relation to other people in the neighbourhood (poor, average or well-off).

Probable CMD was evaluated using the 10-item Kessler Psychological Distress scale (K10) [13], with three additional questions on suicidality. The K10 is a widely used tool to assess non-specific psychological distress in the past month [13]. Each item is rated from 1–5, mainly based on the persistence of a specific symptom—none of the time, a little of the time, some of the time, most of the time, and all of the time. The total score for the 10-item scale is 50. The level of mental distress is then categorized into four groups: Those scoring 10–19 are likely to be well; those scoring 20–24 are likely to have mild mental disorder; those with a score of 25–29 are likely to have a moderate mental disorder; those scoring 30–50 are likely to have severe mental disorder [14]. A cut-off score of 19/20 has a sensitivity of 0.71 and a specificity of 0.90 in relation to meeting the criteria for anxiety and affective disorders according to the Composite International Diagnostic Interview [15]. Both the 10- and 6-item versions of the scale have been validated in Ethiopia among postnatal women, with the 10-item version showing superior validity [16]. We used the validated Amharic (the official language of Ethiopia) version of the K10 [16]. In this postnatal sample the sensitivity and specificity of the K10 were 0.78 and 0.84 respectively. The additional questions about suicide asked interviewees whether they had 1) experienced a death wish; 2) suicidal thoughts; and 3) attempted suicide in the previous 30 days. These three all together defined suicidal behavior.

Screening for alcohol use employed the Fast Alcohol Screening Test (FAST) derived from the Alcohol Use Disorder Identification Test (AUDIT) [17,18]. The FAST questionnaire has only four items and can be completed in just a minute. A total score of 3 or more confirms the occurrence of hazardous alcohol use [19], which was also what defined hazardous use in this study. The FAST has better psychometric properties than the CAGE [20,21], with sensitivity of 0.93 and specificity of 0.88 [19], and comparable to the AUDIT [22]. It is also reported to have a higher sensitivity and specificity than the AUDIT when used in emergency departments [23]. Although not validated in the Ethiopian setting, the AUDIT has been used in neighbouring East African countries [24,25]. Local alcoholic beverages were converted into standard equivalent alcohol units [26].

Experience of stressful life events during the six months period prior to assessment and social support were assessed using the List of Threatening Experiences (LTE) [27] and the Oslo 3-item Social Support Scale (OSS) [28] respectively. The LTE contains 12 categories of significant life events, for example relating to death of close persons, loss of relationships, imprisonment, and being the victim of theft. These 12 categories accounted for two thirds of all events collected in the original development of the tool. The LTE has good test-retest reliability (Kappa: 0.61-0.87) and predictive validity [29]. The OSS contains three items assessing the number of close confidants, perceived level of concern from others and perceived ease of getting help from neighbours. Based on the raw scores, the scale allows a summary score (range 3–14) or categories of social support (strong, average and poor) to be generated. The OSS has good convergent and predictive validity [30,31].

### Administration of assessment instruments

Assessment instruments were administered by trained community health workers in Amharic, the local language of the district. These health workers were high school graduates with one year of training in health care. They were trained for two days and the instruments were piloted and pre-tested in selected sub-districts. The data collection was supervised by nurses and data supervisors with many years of experience in administering a range of mental health-related instruments.

### Data management and analyses

Data were entered into Epi-data version 3.1 and analysed using the Statistical Packages for Social Sciences, version 20 (SPSS 20; IBM Corp 2012). Simple descriptive analyses were used to summarise the profile of the outcomes and factors. Logistic regression models were fitted to assess the association of the two main outcomes (CMD and suicidal behaviour evaluated one at a time) with potential risk factors. These potential risk factors were selected a priori based on evidence from existing literature and our theoretical assumption that these factors would be relevant for the outcomes of interest. Analyses of associations for CMD focused on moderate and severe disorder. Association for suicidal behaviour focused on persistent death wish, frequent suicidal ideation (occurring for at least 50% of time) and suicide attempt. Only factors that were associated with the particular outcome (CMD or suicidality) in the univariate models were included in the corresponding multivariable models in order to limit the potential risk of over-adjusting without compromising identification of potential predictors for each outcome. Most of the variables were analysed as set in the original data collection tools, except for the main psychosocial factors (life events and social support). Thus experience of life events were grouped into three categories (none; 1–2 life events and 3 and above). The total social support scores were re-categorised as per the recommended classes of poor, moderate and strong social support. Additionally, the individual social support domains were entered into the model separately. A main category of formal and informal education (those without formal schooling) were included to take into account the large number of people in Ethiopia who are literate (are able to read and write) through various educational routes, such as religious programmes and the governmental literacy programmes.

### Ethical considerations

The study was approved by the Scientific Committee of the Department of Psychiatry, Addis Ababa University, and the Institutional Review Board of the College of Health Sciences of Addis Ababa University. The conduct of the study was consistent with the Declaration of Helsinki (http://www.wma.net/en/30publications/10policies/b3/). In all cases, informed consent was sought after adequate information about the study, and the potential benefits and risks, had been provided. Participants who had significant level of depression or were suicidal were assessed by a psychiatric nurse and psychiatry residents. Whenever required, treatment was offered to these free of charge.

## Results

### Demographic characteristics

A total of 1497 people were interviewed. Most participants were from the Gurage ethnic group (94.6%), resided in rural villages (90.7%) and were of Orthodox Christian religion. About half (50.4%) were women. The median age of participants was 35.0 years (interquartile range, 27.8 - 45.0) with 8.2% aged 60 or above. Over half of the participants were either illiterate (43.1%) or had not received any formal education although were able to read and write (23.9%). Details are provided in Table 2.

**Table 2:** Socio-demographic characteristics of participants (n = 1497)

**Characteristics**		**Number (%)**
**Sex (n = 1497)**	Male	743 (49.6)
	Female	754 (50.4)
**Age categories (years) (n = 1483)**	<25	226 (15.2)
	25-34	422 (28.5)
	35-44	392 (26.4)
	45-59	321 (21.6)
	60 & above	122 (8.2)
**Age (years) (n = 1483)**	Mean (SD)	37.7 (13.5)
**Marital status (n = 1484)**	Married	1110 (74.8)
	Single	255 (17.2)
	Formerly married*	75 (5.1)
**Ethnicity (n = 1490)**	Gurage	1409 (94.6)
	Others**	81 (5.4)
**Religion (n = 1441)**	Orthodox Christian	1327 (92.1)
	Protestant	80 (5.6)
	Muslim	33 (2.3)
**Education (n = 1375)**	Non-literate	592 (43.1)
	Literate but no formal education	328 (23.9)
	Formal education	455 (33.1)
**Occupation (n = 1436)**	Housewife	404 (28.1)
	Farmer	770 (53.6)
	Private business	130 (9.1)
	Other***	132 (9.1)
**Residence (n = 1486)**	Urban	139 (9.4)
	Rural	1347 (90.7)
**Perceived relative wealth (n = 1458)**	Poor	498 (34.2)
	Average	793 (54.4)
	High	167 (11.5)

*Divorced/widowed/separated; **Amhara, Oromo, Tigre; ***Civil servant and students.

### Psychosocial factors, common mental disorders and suicidal behaviour

Major stressful life events experienced in the previous six months were common and reported by 44.7% of the interviewees. Of these 28.6% had experienced one or two life events while 16.1% had experienced three or more life events. Only 11.9% reported strong social support, while 41.8% reported poor social support (Table 3). Nearly half reported they were either uncertain about the concern that others show towards them or that concern from others was non-existent (49.4%) while 55.9% reported that they find it difficult to get help from others. The overall prevalence of probable CMD was 27.9% (95% CI = 25.6, 30.2), mostly mild (13.9%) or moderate (8.9%) in severity; 5.0% had severe CMD (Table 2). The prevalence (95% CI) of death wish, suicidal ideation, persistent suicidal ideation and actual suicide attempts, constituting suicidal behaviour in the last one-month, were found in 20.5% (18.5, 22.7), 13.5% (11.8, 15.3), 3.3% (2.4, 4.3) and 1.4% (0.9, 2.1) respectively. About a fifth (22.4%) reported hazardous use of alcohol, higher among men (33.4%) compared to women (11.3%). This difference was statistically significant: *X*^*2*^(1) = 62.1; p < 0.001.

**Table 3:** Distribution of adverse life experiences, social support and psychological distress

**Characteristics**		**Number (%)**
Number of stressful life events* (N = 1449)	None	801 (55.3)
	1 or 2	415 (28.6)
	3 and above	233 (16.1)
**Social support (N = 1412)**	Poor	590 (41.8)
	Moderate	654 (46.3)
	Strong	168 (11.9)
**Common mental disorder (N = 1475)**	Likely well	1064 (72.1)
	Mild disorder	205 (13.9)
	Moderate disorder	132 (8.9)
	Severe disorder	74 (5.0)
**Hazardous use of alcohol (N = 1382)**	None	1070 (77.4)
	Yes	312 (22.6)
**Suicidality (in previous 30 days)**	Wish to die (N = 1491)	306 (20.5)
	Suicidal ideation (N = 1497)	202 (13.5)
	Persistent death wish (N = 1444)	47 (3.3)
	Suicide attempt (N = 1493)	21 (1.4)

*Also called threatening life experiences by the developers of the instrument.

### Factors associated with common mental disorder and suicidal behaviour

Both univariate (Tables 4 and 5) and multivariable (Tables 6 and 7) models are presented. In the multivariable model, factors associated with CMD were increasing age (OR = 1.01; 95% CI = 1.00, 1.03; p = 0.035), loss of marriage (OR = 2.34; 95% CI = 1.20, 4.58; p = 0.013), experience of threatening life events in the previous six months (OR = 1.49; 95% CI = 1.01, 2.20; p = 0.046), and hazardous use of alcohol (OR= 1.92; 95% CI=1.39, 2.67; p<0.001. Experiencing one or two life events increased the odds of CMD about four fold (OR = 3.74; 95% CI = 2.42, 5.78; p < 0.001) while experiencing three or more life events doubled the odds to over eight fold (OR = 8.90; 95% CI = 5.57, 14.21; p < 0.001).

**Table 4:** Univariate analysis of factors associated with common mental disorder

		**Well or only mild disorder n%**		**Moderate or severe disorder n%**		**Odds ratio**	**95% CI**		**p value**
		**N%**		**N%**					
**Sex**									
	Male	631	49.7	105	51.0	1.0			
	Female	638	50.3	101	49.0	0.96	0.76	1.20	0.689
**Residence**									
	Urban	115	9.1	22	10.7	1.0			
	Rural	1144	90.9	184	89.3	1.19	0.74	1.93	0.481
**Marital status**	Single	222	17.6	31	15.3	1.0			
	Married	952	75.5	141	69.5	1.04	0.69	1.57	0.855
	Formerly married	85	6.8	31	15.3	2.46	1.44	4.26	0.001
**Ethnicity**									
	Gurage	1199	95.0	190	92.2	1.0			
	Other	63	5.0	16	7.8	0.62	0.35	1.10	0.105
**Religious affiliation**									
	Orthodox	1125	92.1	184	92.9	1.0			
	Other	97	7.9	14	7.1	0.85	0.48	1.52	0.578
**Occupation**									
	House wife	346	28.5	54	26.9	1.0			
	Farmer	646	53.2	114	56.7	0.91	0.52	1.60	0.749
	Private	112	9.2	14	7.0	1.03	0.61	1.74	0.910
	Other	111	9.1	19	9.5	0.73	0.35	1.53	0.404
**Age**									
	Median (IQR)	35.0 (27.8, 45.0)			38.0 (29.1, 50.0)		1.01	1.03	0.002
**Relative wealth**									
	Better off	149	12.0	17	8.6	1.0			
	Average	689	55.6	93	47.0	1.19	0.69	2.05	0.539
	Poor	401	32.4	88	44.4	1.97	1.14	3.42	0.016
**Education**									
	Formal education	391	33.7	58	30.2	1.0			
	Literate but no formal education	270	23.3	53	27.6	1.32	0.88	1.98	0.174
	Non-literate	500	43.1	81	42.2	1.09	0.76	1.57	0.633
**Mental illness in family**									
	None	978	80.7	138	70.8	1.0			
	Yes	234	19.3	57	29.2	1.73	1.23	2.43	0.002
**Life events**									
	None	746	60.8	48	23.6	1.0			
	≤2 events	332	27.1	74	36.5	3.65	2.53	5.27	<0.001
	>2 events	148	12.1	81	39.9	8.25	5.57	12.22	<0.001
**Social support**									
	Strong	152	12.6	15	7.9	1.0			
	Average	555	46.2	88	46.3	1.65	0.93	2.94	0.088
	Poor	495	41.2	87	45.8	1.79	1.00	3.18	0.048
**Can count on others**									
	At least on 1 person to count on	1164	86.9	175	86.2	1.0			
	None	90	7.2	28	13.8	2.07	1.32	3.26	0.002
**Concern from others**									
	At least some concern	616	50.6	80	41.7	1.0			
	Little or uncertain	601	49.4	112	58.3	1.44	1.05	1.95	0.022
**Ease of help**									
	Easy	556	44.1	77	37.7	1.0			
	Difficult	704	55.9	127	62.3	1.30	0.96	1.77	0.088
**Harmful use of alcohol**									
	None	989	80.2	137	67.8	1.0			
	Yes	244	19.8	65	32.2	1.92	1.39	2.67	<0.001

**Table 5:** Univariate model of factors associated with suicidality

		**Non-suicidal**		**Suicidal**		**Odds ratio**	**95% CI**		**p value**
		**n**	**%**	**n**	**%**				
**Sex**									
	Male	627	50.3	111	46.3	1.0			
	Female	620	49.7	129	53.8	1.18	0.89	1.55	0.253
**Residence**									
	Urban	118	9.5	20	8.4	1.0			
	Rural	1119	90.5	219	91.6	0.87	0.53	1.42	0.569
**Marital status**									
	Single	231	18.7	22	9.2	1.0			
	Married	916	74.2	186	77.8	2.13	1.34	3.39	0.001
	Formerly married	88	7.1	31	13.0	3.70	2.03	6.73	<0.001
**Ethnicity**									
	Gurage	1176	94.8	225	93.8	1.0			
	Other	64	5.2	15	6.3	0.82	0.46	1.46	0.493
**Religious affiliation**									
	Orthodox	1109	92.0	211	93.4	1.0			
	Other	97	8.0	15	6.6	0.81	0.46	1.43	0.471
**Occupation**									
	House wife	338	28.4	65	27.7	1.0			
	Farmer	635	53.3	129	54.9	0.95	0.56	1.62	0.858
	Private	110	9.2	19	8.1	1.01	0.61	1.65	0.980
	Other	109	9.1	22	9.4	0.86	0.44	1.67	0.648
**Age**									
	Median (IQR)	35.0(27.0, 45.0)		38.0 (30.0, 50.0)		1.02	1.01	1.03	<0.001
**Relative wealth**									
	Better off	147	12.1	80	7.6	0.49	0.28	0.83	0.008
	Average	620	55.2	119	50.2	0.70	0.52	0.94	0.019
	Poor	396	32.6	199	42.2	1.0			
**Education**									
	Formal education	404	35.4	48	21.2	1.0			
	Able to read/write	261	22.9	65	28.8	2.10	1.40	3.14	<0.001
	Not literate	475	41.7	113	50.0	2.00	1.39	2.88	<0.001
**Mental illness in family**									
	None	959	80.3	167	74.6	1.0			
	Yes	236	19.7	57	25.4	1.39	0.99	1.93	0.054
**Life events**									
	None	736	61.0	62	26.6	1.0			
	≤2 events	320	26.5	91	39.1	3.38	2.38	4.78	<0.001
	>2 events	151	12.5	80	34.3	6.29	4.32	9.15	<0.001
**Social support**									
	Strong	141	12.0	26	11.5	1.0			
	Average	552	46.9	98	43.2	0.84	0.62	1.13	0.245
	Poor	485	41.2	103	45.4	0.87	0.54	1.39	0.555
**Can count on others**									
	At least on 1 person to count on	1138	92.4	211	88.7	1.0			
	None	94	7.6	27	11.3	1.55	0.99	2.44	0.058
**Concern from others**									
	At least some concern	606	50.8	98	43.0	1.0			
	Little or uncertain	587	49.2	130	57.0	1.37	1.03	1.82	0.031
**Ease of help**									
	Easy	546	44.1	90	38.0	1.0			
	Difficult	693	55.9	147	56.9	1.29	0.97	1.71	0.083
**Hazardous alcohol use**									
	None	959	79.3	180	76.3	1.0			
	Yes	251	20.7	56	23.7	1.19	0.85	1.65	0.305

**Table 6:** Multivariable model of factors associated with common mental disorder

		**Odds ratio**	**95% CI**		**p value**
***Demographic Factors***					
**Age**		1.01	1.00	1.03	0.035
**Marital status**					
	Single	1.0			
	Married	0.99	0.59	1.64	0.954
	Formerly married	2.34	1.20	4.58	0.013
***Economic Factors***					
**Relative wealth**					
	Better off	1.0			
	Average	0.76	0.41	1.41	0.391
	Poor	1.23	0.66	2.29	0.524
***Psychosocial factors***					
**Life events**					
	None	1.0			
	≤2 life events	3.74	2.42	5.78	<0.001
	>2 life events	8.90	5.57	14.21	<0.001
**Social support**					
	Strong	1.0			
	Average	0.77	0.40	1.48	0.432
	Poor	0.96	0.66	1.39	0.834
**Can count on others**					
	At least 1 person to count on	1.0			
	None	0.97	0.55	1.71	0.920
**Concern from others**					
	At least some concern	1.0			
	Little or uncertain	1.36	0.95	1.94	0.092
***Clinical factors***					
**Family history of illness**					
	No	1.0			
	Yes	1.09	0.69	1.72	0.920
**Harmful use of alcohol**					
	None	1.0			
	Yes	1.49	1.01	2.20	0.046

**Table 7:** Multivariable model of factors associated with suicidality

		**Odds ratio**	**95% CI**		**p value**
***Demographic Factors***					
**Age**		1.01	1.00	1.03	0.142
**Marital status**					
	Single	1.0			
	Married	1.95	1.01	3.77	0.046
	Formerly married	1.42	0.60	3.39	0.430
**Education**					
	Formal education	1.0			
	Able to read/write	2.06	1.15	3.70	0.016
	Non-literate	2.29	1.33	3.93	0.003
***Psychosocial factors***					
**Life events**					
	None	1.0			
	≤2 life events	2.41	1.53	3.80	<0.001
	>2 life events	2.84	1.67	4.82	<0.001
***Clinical factors***					
**Family history of illness**					
	No	1.0			
	Yes	1.48	0.91	2.40	0.111
**Severity of CMD**					
	None	1.0			
	Mild	6.38	3.97	10.24	<0.001
	Moderate	14.54	8.56	24.70	<0.001
	Severe	60.91	28.01	132.48	<0.001

CMD = common mental disorder.

Marital status, lower educational status, experience of stressful life events and CMD were all associated independently with suicidal behaviour. Thus being married (OR = 1.95; 95% CI = 1.01, 3.77; p = 0.046), being non-literate (OR = 2.29; 95% CI = 1.33, 3.93; p = 0.003) or not having formal education (OR = 2.06; 95% CI = 1.15, 3.70; p = 0.016), approximately doubled the odds of exhibiting suicidal behaviour. Both life events and CMD showed an increase in odds of suicidal behaviour with increase in frequency of life events as well as increase in the severity of the CMD, as shown in Table 7.

## Discussion

Indicators of high level of population level distress were found in this rural district manifested in terms of relatively high levels of CMD, suicidal behaviour, and threatening life events occurring in the context of low levels of reported social support. In relation to CMD, the prevalence found in our study is overall comparable to what has been reported in Ethiopia albeit on the higher margins. For ease of comparison, studies of CMD that have been conducted in Ethiopia are summarised in Table 1. These studies may be categorised into three generations based on the method of case detection and case definition. Case detection in the first generation studies was based on interview by psychiatrists and the studies were conducted in healthcare facilities and in small communities. The facility-based studies reported the prevalence of “conspicuous psychiatric morbidity” to be 16% to 18%. The prevalence from the community-based studies was relatively low, around 6%. The second generation studies used screening tools administered by lay interviewers, mainly the Self Reporting Questionnaire (SRQ) [32]. The SRQ was developed specifically for use in low and middle income countries and does not have a recommended cut-off for defining “caseness”. In these studies of CMD that used the SRQ in Ethiopia, the cut-off values for caseness varied from 5/6 to 11/12. The prevalence of CMD varied from between 5% to about 30% [1,33-39]. Generally studies with lower prevalence of CMD used lower cut-offs. Although the use of such varied cut-offs makes interpretation more difficult, the use of screening tools enables larger populations to be studied. Thus two of the second generation studies evaluated over 10,000 people [33,37]. The third generation studies have used diagnostic assessments, mainly the lay interviewer-administered Composite International Diagnostic Interview [40]. These studies were able to describe the prevalence of various individual conditions that overlap with the concept of CMD (mainly anxiety disorders, depression, somatoform disorders). The prevalence of these disorders varied between 2% and 20% [41-47]. The use of a diagnostic instrument was perhaps a major step forward.

Although our study looked at the prevalence of CMD, the primary objective of our study was to make a determination of the population level mental distress for the provision of a community level intervention. In this context, our study is the only one in Ethiopia that brings together broad population level distress indicators (sucidiality and alcohol use disorder) [48] including CMD and psychosocial stressors. Within the context of CMD studies over the past 40 years, our study is also meaningful given the changes in the country in the past 15 years and the need to understand the impact of these changes on mental health. It is notable, however, that the prevalence of CMD is generally comparable to the older studies from over 15 years ago.

In the context of what is known about CMD in Ethiopia, our study indicates that CMD are relevant public health concerns. At present, the national plan for the scale up of mental health care understandably focuses on more severe disorders (psychosis, major depressive disorder and epilepsy) [49]. Our study indicates that CMD and associated suicidal behaviour are common and need to be considered in the provision of care although the approach may need to include interventions at the population level to address population level determinants.

Suicidal behaviour and psychosocial factors related to mental disorders and suicidal behaviour have not been explored in any detail in Ethiopia. Two studies looked at the lifetime prevalence of suicide attempt, which varied between 1% and 3% [50,51]. For the most part the exploration of psychosocial factors was limited to socio-demographic factors. In line with previous findings, this PRIME study found lower level of education, life events and mental disorder were associated with suicidality [52-54]. The association of married status with suicidality was not in agreement with other studies, for example that of the World Mental Health Survey [54]. We did not explore the reasons for this association. However, one potential explanation is conflicts within marriage or love relations, which are reported to be common psychosocial factors identified in people attempting suicide in Africa [50,51,55,56]. Further replication of this finding and exploration of potential factors that may increase the risk of suicidal behaviour in the African context is required.

The main factors associated with CMD were psychosocial stressors (stressful life events), which was in the expected direction. Loss of a marriage was associated with CMD as reported elsewhere in Africa [57,58] as was increasing age [57,59]. Although low social support was associated with CMD only in the univariate model, it was associated with suicidal behaviour in the full model. Low social support is likely to be an important factor in the pathway to CMD as well as suicidality. It is of note that the finding of low social support goes against the general assumption that social support may be better in “developing” countries because of the extended family and community networks [60-62]. Of interest is that the association of relative wealth with both CMD and suicidality observed in the univariate analysis falls away in multivariable analysis, a pattern observed in relation to income in other LMICs [2]. The combination of higher level of negative life events, lower education, and low social support in conjunction with higher level of stigma [63,64], is likely to constitute a toxic milieu for the onset and maintenance of mental distress and suicidal behaviour.

The very high level of association between CMD and suicidality supports the proposal that “the reductionist model”, which views suicidal behaviour in low income countries as an impulsive response to the stresses of life, is not accurate [65,66]. Mental disorder is likely to be an important concomitant of suicidality and should be considered in all individuals presenting with such behaviour. Furthermore, given the high association between suicidal behaviour and CMD, primary care staff training should include training on the assessment of psychosocial needs and the provision of care for all who present to primary care with suicidal behaviour.

Our study indicates the widespread occurrence of psychosocial stressors. Thus addressing CMD and suicidal behaviour is likely to require broader programmes of psychosocial intervention. Despite the concerns about the acceptability of “talking treatments” in developing countries [67], psychosocial interventions have to be developed as part of the larger scale up plan in the country and in the context of district level programmes of intervention such as PRIME. Given the effectiveness of psychosocial interventions [68-73], improving the acceptability of talking treatments through cultural adaptation of content and delivery of interventions would be important.

### Limitations

The study is cross-sectional and we cannot make inferences about causality. Although the main outcome instrument used in this study has been validated in the country, it was not validated in the local setting. However, pretesting of the instrument was carried out in this local setting. The instruments on CMD focus more on frequency of symptoms than clinical relevance (for example level of distress or disability). Although interpretation of the CMD syndrome identified here may appear difficult, evidence suggests that even mild disorders are associated with impairment [74,75]. Therefore, even if the degree of impairment was not specifically assessed, many people identified with CMD are likely to have a degree of impairment [74]. It is also to be noted that most cases of CMD have milder condition; and although the prevalence appears high, not all individuals with CMD would require treatment. Therefore, identifying individuals that are more likely to require and benefit from treatment is necessary. The analyses did not take into account fully the multi-stage nature of the sampling because we did not have a reliable estimate of the size of eligible household members. However, the sampling at the sub-district level was self-weighting given the sampling at the sub-district level was proportional to the number of households in the sub-districts.

## Conclusions

The population level mental distress as indicated by the level of symptoms of CMD, suicidal behaviour, hazardous use of alcohol and the prevalent nature of risk factors for mental distress in the population is high [52,53]. The results of the study argue for the provision of population level interventions to reduce risk factors and to promote wellbeing. The results also suggest that simple psychosocial interventions applicable for this context need to be developed. Although variation in the prevalence of CMD is observed depending on methods and settings [76-82], overall, there is some consistency in the prevalence of CMD over the years.

## Competing interests

The authors declare that they have no competing interests.

## Authors’ contributions

AF, GM, CL and CH developed the study plan. MS and MH supported data collection. AF, GM and CH participated in the data analysis. AF wrote the initial draft. All authors read the manuscripts, provided intellectual input and approved the final draft and submission.

## Pre-publication history

The pre-publication history for this paper can be accessed here:

http://www.biomedcentral.com/1471-244X/14/194/prepub
